# Analysis of wound infections among pediatric patients following the 2023 Türkiye–Syria earthquakes

**DOI:** 10.1007/s00383-024-05755-4

**Published:** 2024-07-17

**Authors:** Edanur Yeşil, Özlem Tezol, Nahida Gokay, Serra Sürmeli Döven, Merve Mısırlıoğlu, Mehtap Akça, Berfin Özgökçe Özmen, Güldane Dikme, Fatma Durak, Mehmet Alakaya, Feryal Karahan, İsa Kıllı, Necdet Kuyucu

**Affiliations:** 1 Department of Pediatrics, Division of Pediatric Infectious Disease, Mersin University Medical Faculty Hospital, Ciftlikkoy, 33120 Mersin, Türkiye; 2 Department of Pediatrics, Mersin University Medical Faculty Hospital, Ciftlikkoy, 33120 Mersin, Türkiye; 3 Department of Pediatrics, Division of Pediatric Nephrology, Mersin University Medical Faculty Hospital, Ciftlikkoy, 33120 Mersin, Türkiye; 4 Department of Pediatrics, Division of Pediatric Intensive Care, Mersin University Medical Faculty Hospital, Ciftlikkoy, 33120 Mersin, Türkiye; 5 Department of Pediatrics, Division of Pediatric Infectious Disease, Hatay City Training and Research Hospital, Hatay, Antakya, Türkiye; 6 Department of Pediatrics, Division of Pediatric Infectious Disease, Mersin City Training and Research Hospital, Toroslar, Mersin, Türkiye; 7 Department of Pediatrics, Division of Pediatric Haematology and Oncology, Mersin University Medical Faculty Hospital, Ciftlikkoy, 33120 Mersin, Türkiye; 8 Department of Pediatric Surgery, Mersin University Medical Faculty Hospital, Ciftlikkoy, 33120 Mersin, Türkiye

**Keywords:** Child, Earthquake, Infection control, Wound infection

## Abstract

**Purpose:**

On February 6, 2023, two earthquakes of magnitude 7.7 and 7.6 occurred consecutively in Turkey and Syria. This study aimed to investigate the predisposing factors for wound infection (WI) and the microbiological characteristics of wounds after earthquake-related injuries.

**Methods:**

This descriptive study evaluated pediatric patients’ frequency of WI, and the clinical and laboratory parameters associated with the development of WI were investigated.

**Results:**

The study included 180 patients (91 female). The mean age of the patients was 123.9 ± 64.9 months and 81.7% (n = 147) of them had been trapped under rubble. Antibiotic treatment to prevent WI had been administered to 58.8% (n = 106) of all patients. WI was observed in 12.2% (n = 22) of the cases. In patients who developed WI, the incidence of exposure to a collapse, crush syndrome, compartment syndrome, multiple extremity injury, fasciotomy, amputation, peripheral nerve injury, thoracic compression, blood product use, intubation, and the use of central venous catheters, urinary catheters, and thoracic tubes were more frequent (p < 0.05). The need for blood product transfusion was associated with the development of WI (OR = 9.878 [95% CI: 2.504–38.960], p = 0.001). The negative predictive values of not developing WI at values of white blood cell count of < 11,630/mm^3^, creatine kinase < 810 U/L, potassium < 4.1 mEq/L, ALT < 29 U/L, AST < 32 U/L, and CRP < 45.8 mg/L were 93.7%, 96.8%, 90.8%, 93.3%, 100%, and 93.5%, respectively. Gram-negative pathogens (81%) were detected most frequently in cases of WI. Seventy-five percent of patients were multidrug- and extensively drug-resistant.

**Conclusion:**

This study leans empirical approach of our disaster circumstances. In cases with risk factors predisposing to the development of WI, it may be rational to start broad-spectrum antibiotics while considering the causative microorganisms and resistance profile to prevent morbidity.

## Introduction

Earthquakes are among the most destructive disasters that cause mass casualties. Turkey has experienced natural disasters throughout its history. On February 6, 2023, consecutive earthquakes with magnitudes of 7.7 and 7.6 Mw on the Richter scale occurred in Kahramanmaraş, a province of Turkey [1]. As a result of those earthquakes, according to official records, more than 50,000 people died, an area of approximately 350,000 km^2^ was affected, more than 150,000 buildings were destroyed, and more than 800,000 buildings were severely damaged [2]. The World Health Organization and the United Nations defined the Türkiye-Syria earthquakes as a Grade 3 emergency because they affected more than one country and required mass support [2, 3].

Individuals exposed to earthquake trauma could experience several types of complications including internal organ injuries, fractures, crush syndrome, and soft tissue infections in the form of wound infection (WI) [4]. Injuries caused by the collapse or crashing of stones, concrete, or rubble could lead to fractures, compartment syndrome, amputation, or fasciotomy and these factors predispose to post-earthquake WI [5–7]. Gram-negative agents such as *Acinetobacter baumannii*, *Pseudomonas aeruginosa*, and *Enterobacteriaceae* spp. and gram-positive agents such as *Staphylococcus aureus* are most often found as the causative agents in post-earthquake wound infections [4–6, 8–10]. Infection of open wounds increases the risk of septicemia and organ dysfunction and may lead to limb loss in cases of trauma-induced tissue ischemia. Debridement of ischemic tissue is useful in preventing subsequent infections [11]. Early antibiotic treatment may also prevent morbidity and mortality by preserving tissue integrity. In addition to WI, post-earthquake tetanus and respiratory tract infections are among the infectious diseases that may develop if not prevented carefully [12].

Although many health and medical support workers traveled to Kahramanmaraş and neighboring provinces after the earthquakes, the disaster, which covered a very large area, disrupted health services. Therefore, the existing capacity was insufficient and many patients were transferred to neighboring hospitals. One of the hospitals to which patients were transferred was our tertiary 680-bed university hospital in our province, which is 300 km away from the epicenter and where there were no casualties due to the earthquakes. The aim of this study is to contribute to the literature by sharing the infection prevention package applied to treat existing infections and prevent the development of secondary infections in earthquake survivors admitted to our hospital (referrals or individuals), to determine the microbiological characteristics of wounds that developed after earthquake trauma, and to investigate risk factors for the development of WI.

## Materials and methods

### Data collection

This retrospective study included pediatric patients admitted to the pediatric emergency and pediatric infectious diseases clinics for earthquake-related injuries in the 1-month period immediately following the February 6, 2023, Kahramanmaraş earthquakes (i.e., between February 6, 2023 and March 6, 2023). The demographic characteristics of the patients, time of admission to the hospital, status of being trapped under rubble, hospitalization, amputation status, fasciotomy, hemodialysis status, and use of blood products (erythrocyte suspension, fresh frozen plasma, or albumin) and invasive methods such as intubation, central venous catheter (CVC), or urinary catheter during hospitalization were recorded. White blood cell (WBC) count, C-reactive protein (CRP), sedimentation level, creatine kinase (CK), urea, creatinine, alanine transaminase (ALT), aspartate aminotransferase (AST), sodium, and potassium (K) levels were recorded. Relatives of patients with soft tissue injuries who were followed as outpatients and those of patients who did not return for follow-up examination after discharge were called by telephone and asked about the patient’s general condition and WI status within the 3 months following the earthquakes. Demographic, clinical, and laboratory data of the patients were retrospectively analyzed from their files in the hospital information management system. The study was approved by the Mersin University Faculty of Medicine’s Clinical Research Ethics Committee with number 2024/339.

### Wound infection management

Tetanus prophylaxis, isolation recommendations in the presence of infection, and antibiotic treatments appropriate to the condition of the wound were applied [13, 14].

#### Infection prevention package

- In cases of skin/tissue injuries after earthquake trauma, decontamination of wounds from foreign bodies, disinfection, and debridement of devitalized tissue were performed as necessary.
- Wounds at risk of tetanus were identified. Accordingly, the following wounds were considered “dirty”: those contaminated with dirt, feces, saliva, or soil; penetrating or puncture wounds; and wounds with necrosis, gangrene, frostbite, or burns. The tetanus vaccine and immunoglobulin were administered according to the immunization statuses of the patients [15].
- Empirical antibiotic treatment options were chosen according to the injury status (Table 1). Antibiotic treatments were administered for 7 days for open wounds of at least 6 h. In patients who were discharged earlier, treatment with oral antibiotics was completed to 7 days. In patients with infection, the duration of treatment was determined according to the wound healing status. In patients hospitalized in the wards, wound status was checked daily and cultures were taken in the presence of purulent drainage and when infected tissue was seen during debridement. For infections occurring 48 h after hospitalization, nosocomial pathogens were considered in empirical treatment. Nephrotoxic antibiotics were avoided in patients with crush syndrome and those receiving dialysis. In the event of renal failure, antibiotic doses were adjusted.
- To prevent possible respiratory infections, patients’ companions were told to wear masks and they received training about appropriate hygienic conditions.

**Table 1:** Patient categories, antibiotic prophylaxis protocols, and development of wound infection

Patient category	n (%)	Antibiotic prophylaxis protocol	Development of wound infection^b^	
			Yes n	No n (%)
Those receiving preoperative prophylaxis for non-open injury	4 (2)	Cefazolin	WI = 0	4 (2)
Those with dirty^a^ open wounds	84 (46)	Ceftazidime + metronidazole	Superficial WI (n = 2) Deep WI (n = 2)	80 (51)
Those with purulent discharge/foul-smelling wounds	11 (6)	Ceftazidime + metronidazole + teicoplanin/vancomycin	Superficial WI (n = 2) Deep WI (n = 9) Surgical site infection (n = 2)	0
Septic/unresponsive to initial treatment	2 (2)	Meropenem + vancomycin/teicoplanin	Deep WI (n = 2)	0
Outpatients	5 (3)	Amoxicillin-clavulanate	WI = 0	5 (3)
Not receiving prophylaxis	74 (41)	–	Superficial WI (n = 1) Deep WI (n = 4) Surgical site infection (n = 1)	69 (44)
Total	180 (100%)	–	22 (12.2%)	158 (87.8%)

### Definitions

#### Wound infection

WI was diagnosed in accordance with the Centers for Disease Control and Prevention (CDC) skin and soft tissue infections surveillance criteria. Surgical site infection (SSI) was defined in accordance with the CDC diagnostic criteria. Patients with SSI were also included among WI patients. WIs were categorized as superficial (skin/subcutaneous) WI and deep (fascia/muscle) WI [16].

#### Crush syndrome

Patients with crushing injuries and myoglobinuria and/or hematuria, renal failure, and CK levels of > 1000 U/L were diagnosed with crush syndrome [16].

### Microbiological method

Tissue/wound or pus cultures were obtained from patients with suspected WI. Cultures were taken by swab (Amies transport medium) or as tissue material and transferred to a microbiologist. Manual biochemical tests were performed for bacterial identification in tissue cultures. First, the samples were inoculated on 5% sheep blood agar, eosin methylene blue agar, or chocolate agar. After 24–48 h of incubation, Gram staining was performed and gram-positive cocci were identified by catalase, coagulase, DNase test, and hemolysis ability while gram-negative bacilli were identified by Krigler iron agar, motility, indole test, citrate, and urea metabolism. Most isolates were reevaluated with the VITEK 2 system (BioMérieux, France) for bacterial identification. Antibiotic susceptibility tests were evaluated according to EUCAST criteria. Microorganisms were identified as multidrug-resistant (MDR) or extensively-drug resistant (XDR) according to standardized definitions [17].

### Statistical analysis

IBM SPSS Statistics 25.0 (IBM Corp., USA) was used for statistical analysis. The normality of continuous measurements was checked with histograms and the Shapiro–Wilk test. The Student t-test or Mann–Whitney U test was used to compare continuous variables between the groups of patients with and without WI. Descriptive statistics were given as mean ± standard deviation or median (interquartile range, 25th–75th percentile). Categorical variables were expressed as percentages and numbers, and they were compared between the groups with and without WI using the chi-square test. In order to determine the risk factors for WI, parameters found to be significant by univariate logistic regression analysis (open wound, collapse, crush syndrome, multiple extremity injury, compartment syndrome, fasciotomy, amputation, peripheral nerve injury, thoracic compression, blood product transfusion, intubation, CVC, urinary catheter, thoracic tube, hospitalization, WBC, CRP, ALT, and AST) were included in the model and the backward Wald method was used. The fit of the model for WI parameters to the data was checked with the Hosmer–Lemeshow test (χ^2^ = 4.708; p = 0.696) and good model-data fit was confirmed. Furthermore, the overall correct classification rate of the model was 86.7%. Data were presented with values of odds ratio (OR) and 95% confidence interval (CI). Cut-off points for continuous parameters were obtained using receiver operating characteristic curve analysis for predicting WI. Cut-off points, sensitivity, specificity, and positive and negative predictive values were given as descriptive statistics. Statistical significance was accepted at p < 0.05.

## Results

**Fig. 1:**
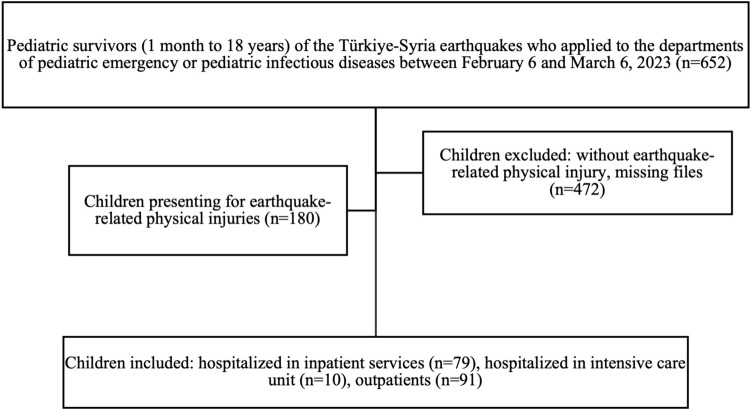
Flowchart of the study population

This study included 180 patients. The study population is shown in Fig. 1. The mean age of the patients was 123.9 ± 64.9 months and 50.6% (n = 91) were female. Patients were most commonly admitted from Hatay province (76.7%, n = 138), and 81.7% (n = 147) of the patients had remained under collapsed rubble for a median of 240 (60–750) minutes. Patients were admitted to our hospital in a median of 3 (2–6.7) days after the earthquake, and 55% (n = 89) were hospitalized for a median of 5.5 (4–9) days. Intensive care unit (ICU) admission was necessary for 5.6% (n = 10) of the patients.

In empirical antibiotic selection, amoxicillin clavulanate was preferred for outpatients and cefazolin was preferred for preoperative prophylaxis in cases of non-open injuries. Ceftazidime and metronidazole were preferred for contaminated/dirty open injuries with soil, feces, or construction materials, and vancomycin/teicoplanin was additionally administered in the presence of purulent discharge and foul odor. Vancomycin/teicoplanin and meropenem were chosen for septic patients and patients who did not respond to initial treatment. Antibiotic treatment was received by 58.8% (n = 106) of the patients and WI was observed in 16% (n = 17) of those who received antibiotic treatment. The remaining 5 patients who did not receive antibiotic treatment but had WI had not been admitted to our hospital before the infections began and presented for the first time with WI.

WI developed in 12.2% (n = 22) of the total patients, 22.4% (n = 20) of the inpatients, and 2.2% (n = 2) of the outpatients. Demographic, clinical, and laboratory data of the patients with and without WI are given in Table 2. Among the patients who developed WI, exposure to a collapse, open wounds, crush syndrome, multiple extremity injury, compartment syndrome, peripheral nerve injury, thoracic compression, fasciotomy, amputation, and the use of CVC, urinary catheter, thoracic tube, and blood product transfusion were observed more frequently (p < 0.05). Hospitalization/ICU admission was more frequent and length of stay was longer in patients with WI (p < 0.05). WBC count, CRP, ALT, AST, and CK values were higher in patients with WI (p < 0.05).

**Table 2:** Demographic, clinical, and laboratory characteristics by the development of wound infection groups

Variable	WI (n = 22)	Non-WI (n = 158)	p
Age, months, mean ± SD	145 ± 57.6	120.9 ± 65.5	0.104

	% (n)		
Sex, male	36.4 (8)	51.3 (81)	0.279
Open wound	100 (n = 22)	61 (75)	**0.0001**
Trapped under rubble	100 (22)	79.1 (125)	**0.016**
Time spent under rubble, minutes	390 (120–1980)	240 (48–720)	0.457
Empirical antibiotic intake	77.3 (17)	56.3 (89)	0.101
Crush syndrome	72.7 (16)	27.8 (44)	**0.0001**
Fracture	40.9 (9)	27.8 (44)	0.465
Multiple extremity injuries	31.8 (7)	9.4 (15)	**0.011**
Compartment syndrome	27.3 (6)	7 (11)	**0.008**
Fasciotomy	18.2 (4)	4.4 (7)	**0.031**
Amputation	18.2 (4)	2.5 (4)	**0.009**
Intracranial hemorrhage	40.9 (9)	21.5 (34)	0.083
Peripheral nerve injury	40.9 (9)	6.3 (10)	**0.0001**
Pneumothorax/pneumomediastinum	13.6 (3)	3.8 (6)	0.082
Thoracic compression	22.7 (5)	5.1 (8)	**0.012**
Vertebral column injury	13.6 (3)	4.4 (7)	0.108
Pulmonary contusion	9.1 (2)	3.2 (5)	0.205
Blood product use (ES/FFP/albumin)	54.5 (12)	8.2 (13)	**0.0001**
Intubation	18.2 (4)	2.5 (4)	**0.009**
Central venous catheter	36.4 (8)	13.3 (21)	**0.011**
Urinary catheter	59.1 (13)	28.5 (45)	**0.008**
Thoracic tube	18.2 (4)	1.3 (2)	**0.002**
Hemodialysis	22.7 (5)	11.4 (18)	0.167
Hospitalization	90.9 (20)	43.7 (69)	**0.0001**
ICU hospitalization	22.7 (n = 5)	3.2 (5)	**0.003**
Length of hospitalization (days)	10 (7–20.2)	5 (4–7.2)	**0.004**

	Mean ± SD		
Hgb (g/dL)	10.9 ± 3.7	12.0 ± 2.2	0.202

	Median (25^th^–75th percentiles)		
WBC (× 10^3^/µL)	14,800 (10,950–19155)	10,740 (8605–15212)	**0.017**
Platelets (× 10^3^/µL)	286,000 (191,000–399500)	302,500 (246,000–389750)	0.240
CRP (mg/L)	75 (21–1139)	14.2 (3.7–46)	**0.025**
Sedimentation (mm/hour)	21 (13–44)	25 (25–53)	1.000
ALT (U/L)	122 (31–212)	24 (16–94)	**0.012**
AST (U/L)	199 (48–825)	43 (24–164)	**0.023**
Urea (mg/dL)	32.2 (23–65)	27.8 (20–34)	0.810
Creatinine (mg/dL)	0.4 (0.3–1.3)	0.37 (0.3–0.5)	0.328
Sodium (mEq/L)	131 (129–137)	136 (134–138)	0.236
Potassium (mEq/L)	4.2 (3.7–4.9	3.9 (3.6–4.2)	0,499
CK (U/L)	12,234 (1500–96449)	396 (120–6716)	**0.001**

Statistically significant data with p < 0.05 are indicated in bold

*WI* wound infection, *SD* standard deviation, *ES* erythrocyte suspension, *FFP* fresh frozen plasma, *ICU* intensive care unit, *Hgb* hemoglobin, *WBC* white blood cell count, *CRP* C-reactive protein, *ALT* alanine transaminase, *AST* aspartate aminotransferase, *CK* creatinine kinase

When the factors associated with the development of WI were analyzed, it was found that the need for blood products increased the risk of developing WI 9.8-fold (OR = 9.878 [95% CI: 2.504–38.960], p = 0.001).

**Fig. 2:**
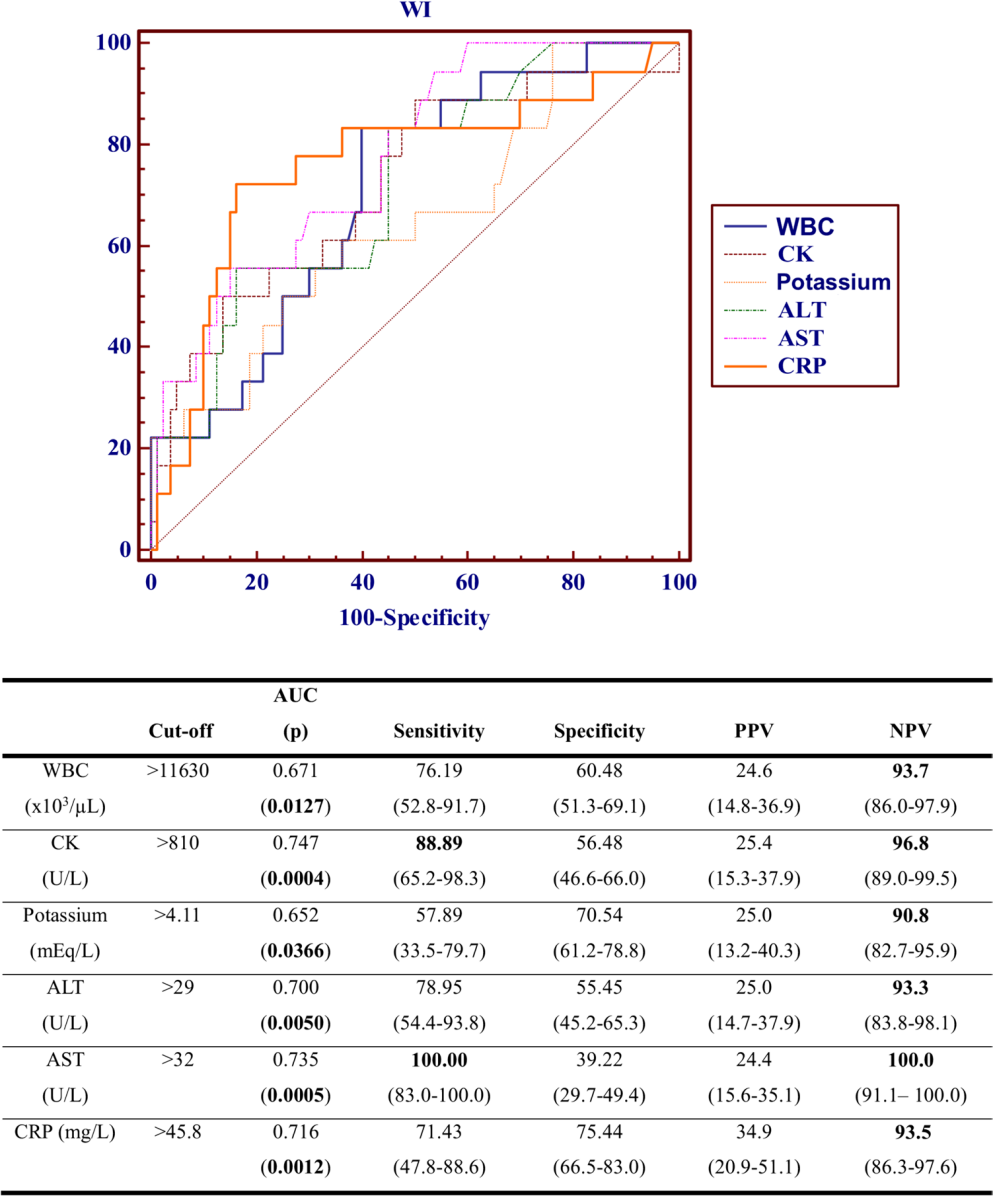
Sensitivity and specificity of potential laboratory parameters for development of wound infection. *WBC* white blood cell count, *CK* creatinine kinase, *ALT* alanine transaminase, *AST* aspartate aminotransferase, *CRP* C-reactive protein, *AUC* Area under the curve, *PPV* positive predictive value, *NPV* negative predictive value

The negative predictive values of WI were 93.7%, 96.8%, 90.8%, 93.3%, 100%, and 93.5% in patients with WBC count of < 11,630/mm^3^, CK < 810 U/L, potassium < 4.1 mEq/L, ALT < 29 U/L, AST < 32 U/L, and CRP < 45.8 mg/L, respectively (Fig. 2).

### Characteristics of patients with wound infections

All of the patients with WI had been trapped under rubble and had open wounds. Among these patients, 90.9% (n = 20) were hospitalized for a median of 10 (7–20.2) days. Five patients were followed in the ICU. Twenty-three percent (5/22) of all WIs were superficial and 77% (17/22) were deep WIs. Of the 37 tissue/wound/pus cultures obtained from 22 patients with WIs, 21 (56.7%) had growth in culture. The growth was polymicrobial in 52.3% (11/21) of the cultures and a total of 32 microorganisms were detected (Table 3). Gram-negative (81%, n = 26) followed by gram-positive (19%, n = 6) microorganisms were the most common pathogens. Among these, *Enterobacteriaceae* spp. were the most common pathogens (40.5%, n = 13), followed by *Acinetobacter baumannii* (25%, n = 8) and *Pseudomonas aeruginosa* (15.6%, n = 5). Fifty percent (3/6) of the gram-positive pathogens were methicillin-sensitive *Staphylococcus aureus* (MSSA) and the rest were methicillin-resistant coagulase-negative staphylococci (MRCNS). Seventy-five percent of the isolates were MDR and XDR isolates and their antibiogram characteristics are given in Table 3. Five of the *A. baumannii* isolates were XDR and all of them were colistin-sensitive. The obtained *Enterobacteriaceae* spp. were susceptible to meropenem except for one isolate. All *P. aeruginosa* isolates were susceptible to meropenem. Two patients developed bacteremia secondary to soft tissue infection.

**Table 3:** Microorganisms detected from wound, pus or tissue cultures and their resistancy profiles

Groups	% (n)	Species	(n)	Resistant isolates										
				Amicasin	Amoxycillin+clavulanic acid	Ampicillin	Cefepime	Cefoxitin	Ceftazidime	Ceftiaxone	Ciprofloxacin	Clindamycin	Erithromycin	Ertapenem
Non-fermentative gram-negative rods	40.5 (13)	*Acinetobacter baumanniae*	8	7							8			
		*Pseudomonas aeruginosa*	5	0			4		4		4			
Fermentative gram-negative rods	40.5 (13)	*Proteus mirabilis*	4	1	0	3	0		0	0	0		0	0
		*Klebsiella oxytoca*	3	0	0	3	0		0	3	0			0
		*Enterobacter cloaca*	2	0	2	2	2		2	2	0			2
		*Escherichia coli*	2	2	0	2	2		2	2	1			0
		*Klebsiella pneumoniae*	1	0	0	1	0		0	0	1			0
		*Enterobacter hormaechei*	1	0	1	1	1	1	1	1	0			1
Gram-positive bacteria	19 (6)	MSSA	3								3	0	0	
		MRKNS	3								4	2	1	

Groups	% (n)	Species	(n)	Resistant isolates										
				Gentamicin	İmipenem	Levofloxacin	Linezolid	Meropenem	Penisicillin G	Ceftazidime/avibactam	Piperacillin Tazobactam	Trimethoprim/sulfamethoxazole	Tobramycin	Kolistin
Non-fermentative gram-negative rods	40.5 (13)	*Acinetobacter baumanniae*	8	8	5	6		5				5		0
		*Pseudomonas aeruginosa*	5		4	4		0		0	4		0	
Fermentative gram-negative rods	40.5 (13)	*Proteus mirabilis*	4	0	4	0		0				4		
		*Klebsiella oxytoca*	3	0	0	0		0				3		
		*Enterobacter cloaca*	2	0	0	0		0				0		
		*Escherichia coli*	2	2	0	0		0				2		
		*Klebsiella pneumoniae*	1	0	0	1		0				1		
		*Enterobacter hormaechei*	1	0	1	0		1				1		0
Gram-positive bacteria	19 (6)	MSSA	3	0		3	0		3			1		
		MRKNS	3	0		3	0		3			1		

*MSSA* methicilline sensitive *Staphylococcus aureus*, *MRKNS* methicilline resistant koagulase negative staphylococcus

Three (9%) of the culture growths occurred 48 h after hospitalization and SSI was diagnosed in those cases. One of these wounds was superficial and the others were deep WIs. Among these three nosocomial isolates, one was XDR *A. baumannii*, one included both *P. aeruginosa* and *E. cloacae*, and one was *P. aeruginosa*. Bacterial growth was detected in 60% (3/5) of patients with WIs hospitalized in the ICU. Two of them were XDR *A. baumannii* and one included *Klebsiella oxytoca* and *Proteus mirabilis*; these grew in three different cultures.

Among the identified cases of WI, 77% (n = 17) occurred in the extremities, 13.6% (n = 3) in the face, and 9% (n = 2) in the lumbosacral area. Two MRCNS isolates were detected in three facial wound cultures. No patients died from among the total of 180 patients. Tetanus was not observed in the follow-up of any patients with soft tissue injuries. When the trauma patients or their families were questioned about the development of soft tissue infections within 3 months after discharge, it was learned that no new infections had developed.

## Discussion

Infections are one of the most common complications in trauma patients. In the literature, there are studies determining the risks of WI, but no other study identified to date has comprehensively addressed the infection protocol, clinical and laboratory parameters, and antibiotic resistance profiles in the form of empirical treatments applied. Determining the risk factors that may cause WI in post-earthquake trauma cases and identifying the causative agents, their resistance profiles, and the precautions that can be taken will be instructive for future earthquakes.

The pathogens identified in our study were quite different from those identified in typical soft tissue infections. In our study, empirical treatment was selected to include gram-negative *Enterobacteriaceae* spp. because gram-negative infections were observed to be prominent after the earthquakes. In a study by Miskin et al., similar antibiotic treatments were utilized [10]. In addition to medical care such as amputation, wound care, flap care, or isolation measures, patients also received physical therapy, rehabilitation, and psychological support.

In our study, it was found that exposure to a collapse, fasciotomy, amputation, and crush syndrome were more common in children who developed WIs after earthquake-related injuries. Similar to our study, WI was more common in children who underwent fasciotomy in the study by Keven et al. [18]. In another study, again similar to ours, exposure to a collapse, fasciotomy, and amputation were more common in patients with WI [7]. In a study evaluating the risk of SSI in patients with multiple extremity damage or bone fractures, no increase in the risk of infection was found [19]. We did not find a relationship between the presence of fracture and WI, but WI was more common in cases with damage to multiple extremities.

In a study evaluating SSIs in patients who underwent fasciotomy due to earthquake injuries, renal insufficiency was found to be associated with infection [19]. In our study, patients with renal insufficiency were included in the group with crush syndrome and it was found that WI developed more frequently among these patients. In the aforementioned study, CK values above 17.839 U/L were associated with the development of SSI [19]. In our study, rhabdomyolysis developed in patients with severe extremity damage, resulting in an increase in CK, ALT, AST, and K values and consequently acute renal failure and crush syndrome. Our findings indicated that the determined cut-off points for CK, ALT, AST, and K had high negative predictive values for WI.

In our study, gram-negative agents were the most common causative agents of WIs. Most of the patients were trapped under collapsed rubble including construction materials and soil or stones. Gram-negative microorganisms can be found in soil and water and are often introduced to wounds caused by earthquake trauma. The fact that the patients could not receive medical assistance in the early period and that interventions such as debridement or wound care for contaminated wounds were only performed after admission to the hospital increased the risk of the contamination of wounds with pathogens. Studies on soft tissue infections due to earthquake trauma have shown that gram-negative microorganisms are predominant [7, 18, 20–24]. In a review of 10 studies examining wound infection isolates seen after earthquakes, it was reported that gram-negative agents were most common (81.6%; 40% non-fermentative, 37.8% fermentative), with 34.4% of the isolates being *Enterobacteriaceae* spp., followed by 20% *Acinetobacter baumannii*. *Staphylococcus aureus* was the most common gram-positive pathogen [10]. These results are similar to those of the present study. *Acinetobacter baumannii* is normally encountered as a hospital-associated infection (HAI) pathogen. In the post-earthquake HAI study of Öncül et al., it was reported that the identified *A. baumannii* isolates were related to the earthquake, although growth occurred 48 h after hospitalization, and *A. baumannii* isolates were found less frequently in hospital surveillance [5]. The fact that *Acinetobacter baumannii* has been reported as the leading causative pathogen in most of these studies suggests that it can easily grow in tissues with ischemia or extensive trauma exposure and that it is associated with contamination from earthquakes, not hospitals.

In our study, gram-positive microorganisms were predominant in cultures obtained from facial regions. Facial injuries were superficial and contamination with skin flora was thought to be highly probable. In the study conducted by Liu et al., gram-positive agents were found more frequently in scalp injuries [25]. In the study by Miskin et al., gram-negative microorganisms were more common in areas other than the face [10].

In our study, 52.3% (11/22) of the cultured growths were polymicrobial, which is similar to the rate of polymicrobial WIs reported after an earthquake in Pakistan (59.6%) [20]. Following a tsunami in Thailand in 2004, 71.8% polymicrobial growth was detected, and this rate was 67.7% after the Wenchuan earthquake in China in 2008 [8, 26]. Polymicrobial growths can be encountered as the colonization of more than one agent with subsequent infection, especially in cases of dirty injuries contaminated by soil from outdoor environments.

In our study, MDR and XDR isolates constituted 75% of all isolates. In the 2005 Pakistan earthquake, Kiani et al. detected MDR isolates in 61.5% of all samples [20]. Similarly, in other studies, it was observed that the wound infection isolates obtained from earthquake or tsunami victims were mostly gram-negative, polymicrobial, and MDR pathogens [8, 27, 28]. In the study by Öncül et al. of HAIs seen in hospitalized patients after the 1999 Marmara earthquake, it was reported that half of the infections were due to WIs, and the most common isolates were *Acinetobacter baumannii* and these were MDR [5]. In our study, *A. baumannii* strains were susceptible to colistin and *Enterobacteriaceae* spp. isolates were mostly susceptible to meropenem. Considering that cultures obtained from earthquake trauma wounds may be MDR or XDR, it would be rational to determine the effective treatment in a broad spectrum, especially when a specific pathogen is grown and no response to empirical treatment is obtained.

In a study conducted after the 2005 Pakistan earthquake, WIs were observed in 32.7% (56/171) of patients admitted with earthquake trauma [20]. After the 2008 Wenchuan earthquake, the rate of WIs in pediatric patients was 51% (50/98) [8]. After the 2004 Thailand tsunami, skin and soft tissue infections were reported in 66.3% (515/777) cases of traumatic injuries [26]. In our study, the rate of WIs in patients with crush syndrome was 26.6% (16/60). In a previous study investigating infections in patients with crush syndrome, the WI rate was found to be 30.6% [7]. In our study, the infection rate was 36.3% (4/11) in patients who underwent fasciotomy. In an evaluation of SSIs after fasciotomy following the 2023 Kahramanmaraş earthquakes, the rate of fasciotomy infection was found to be 50% (58/116) [19]. Some notes were also compiled regarding empirically initiated treatments in different studies. In one study, first-generation cephalosporin was used for preoperative fasciotomy prophylaxis and for 3 days postoperatively, and in another study, it was stated that most patients received amoxicillin-clavulanate in empirical treatment [19, 26]. The infection rate was found to be higher in those studies. Similar to our study, in the study conducted by Öncül et al., in which ceftazidime and metronidazole or ciprofloxacin and metronidazole were used in empirical treatment, the WI rate was found to be lower compared to those of other similar studies [5]. The low rate of WI in our study can be considered as reflecting the success of the WI management applied for patients during the follow-up period. When the guidelines for skin and soft tissue infections are evaluated, it is seen that treatments for gram-positive agents are most often recommended [29]. Applying the first-line treatments recommended in these guidelines to post-earthquake wounds may lead to treatment failure and morbidities, especially as it is known that colonization by gram-negative polymicrobial agents may occur with a high risk of MDR infections. Therefore, it could be rational to choose empirical treatments appropriate for the mentioned agents for patients injured as a result of earthquake trauma.

In our study, most of the causative agents were microorganisms contaminating wounds caused by earthquake trauma; nosocomial infections were found less frequently. In studies on post-earthquake WI, most infections are seen to occur 48 h after admission and efforts to detect post-traumatic agents are insufficient. Knowing the causative microorganisms that can be seen in earthquake-related wounds would be beneficial in empirical treatment. In our study, empirical treatments and an infection prevention package were initiated in advance, especially for WIs that may develop in the context of ischemia, and this prevented obvious infection findings such as purulent drainage, increased inflammation, and tissue loss and significantly decreased the total WI rate (12.2%) compared to similar studies.

This study involves a diverse cohort of patients, each experiencing varying degrees of individual trauma. The limitation of our study was that the lack of data on factors contributing to wound infections, such as nutritional status, pre-existing medical conditions and duration of exposure. Despite our efforts of categorizing patients, conducting precise statistical comparisons remains challenging. Another limitation was that the medical support provided to the patients from the time they were under collapsed rubble to the time of their admission to the hospital could not be recorded or communicated to us due to the chaotic environment following the disaster. A group of patients applied directly to our hospital from the earthquake zone by their own means. The strength of our study is that it is the first in the literature to examine the risk of WI development in post-earthquake wounds in such detail.

This study primarily relies on empirical observation and approaches, offering significant insights. Its strength lies in the richness of its data, but it does not a definitive guideline or recommendation. All disasters and patient factors should be evaluated in their own circumstances, risk factors should be identified, and empirical treatments should be applied according to those conditions.

In conclusion, although it is our greatest hope that the world will not encounter such a significant disaster again, the infection precaution package that we applied in this study may offer empirical observation as an infection control approach in the event of similar disasters. Considering that gram-negative microorganisms are predominant agents while selecting empirical treatment for post-earthquake injuries, appropriate planning will reduce the likelihood of WIs. In our study, blood product use was found to be a risk factor for WI and it should not be ignored that WI may develop in the presence of WBC, CRP, ALT, AST, AST, K, and CK values increased above the relevant cut-off points.

## Data Availability

All data generated or analysed during this study are included in this published article.
